# Interphase Chromosomes Mingle with Their Peers

**DOI:** 10.1371/journal.pbio.0040174

**Published:** 2006-04-25

**Authors:** Françoise Chanut

Though chromosomes appear as discrete, tidy rod-like bodies with distinct sizes and shapes during cell division, they unravel and morph into what looks like a tangled ball of yarn at the end of each division, when they re-form the cell's nucleus. Nevertheless, experimental evidence from the past 20 years suggests that they remain separate entities throughout the cell cycle. A new study by Miguel R. Branco and Ana Pombo now calls this evidence into question by showing that chromosomal interactions are frequent in the nucleus of human cells during interphase, the part of the cell cycle that lies between cell divisions.

The yarn that fills the interphase nucleus is chromatin, which consists of DNA coiled around histone proteins. In the 1980s, cell biologists developed a technique they termed FISH (for fluorescence in situ hybridization) that allowed them to stain each chromosome a different color. FISH staining showed that even in their unfolded state, chromosomes allow very little—if any—mingling of their chromatin. But FISH requires a harsh chemical treatment that is known to alter chromatin structure. Branco and Pombo developed a modified FISH technique that maintains chromatin integrity and improves the resolution of chromosome visualization. Using this technique, they uncovered more intermingling among interphase chromosomes of human cells than previously observed. Further experiments suggest that intermingling plays an important part in chromosome structure and gene expression.

FISH is normally performed on intact nuclei, to preserve the three-dimensional arrangement of chromosomes. By contrast, Branco and Pombo carried out their staining on cells they had previously frozen and sliced into ultrathin sections. The dyes were able to penetrate the thin samples easily, which eliminated the need for aggressive detergents that would disrupt chromatin organization. The researchers applied various pair-wise combinations of dyes to their ultrathin sections and scored as intermingling any spot of overlapping dye signals. Models based on classic FISH experiments suggest that chromosome territories (CTs), each of which contains the chromatin of a single chromosome, are separated by a protein matrix called an interchromatin domain (ICD.) But Branco and Pombo found that each chromosome mingles on average 2 percent of its chromatin with the chromatin of any other chromosome. Given that human cells contain 23 pairs of chromosomes, this means that intermingling might affect 46 percent of the volume of any chromosome, which poses a severe challenge to the notion of a distinct ICD compartment.

When they examined areas of intermingling at the higher resolution afforded by electron microscopy, Branco and Pombo found that DNA sequences from two mingling chromosomes came into close enough proximity to interact at the molecular level. This observation prompted them to wonder whether intermingling is related to biological functions such as DNA repair and gene expression, both of which rely on the bridging of distant pieces of DNA by large protein complexes.

DNA repair mechanisms are activated when chromosomes break, for instance after prolonged exposure to radiation. When the repair occurs by re-joining the broken ends of two distinct chromosomes, a translocation ensues. In irradiated lymphocytes, translocations occur with various frequencies between various chromosome pairs. Branco and Pombo found a strong correlation between the extent of intermingling and the frequency of translocation for given chromosome pairs. They conclude that intermingling areas are privileged sites for the occurrence of translocations.

To demonstrate a link between chromosome intermingling and gene expression, the researchers inhibited the major lymphocyte's RNA polymerase, one of the enzymes that transcribes DNA sequences into RNAs. Intermingling decreased for some chromosomes and increased for others, confirming that intermingling patterns are molded by a cell's transcriptional activity. Because different cell types express different subsets of genes, intermingling may explain why different cell types are prone to different chromosomal re-arrangements. For more on visualizing chromosomal territories, see the related Primer (DOI: 10.1371/journal.pbio.0040155).

## 

**Figure:**
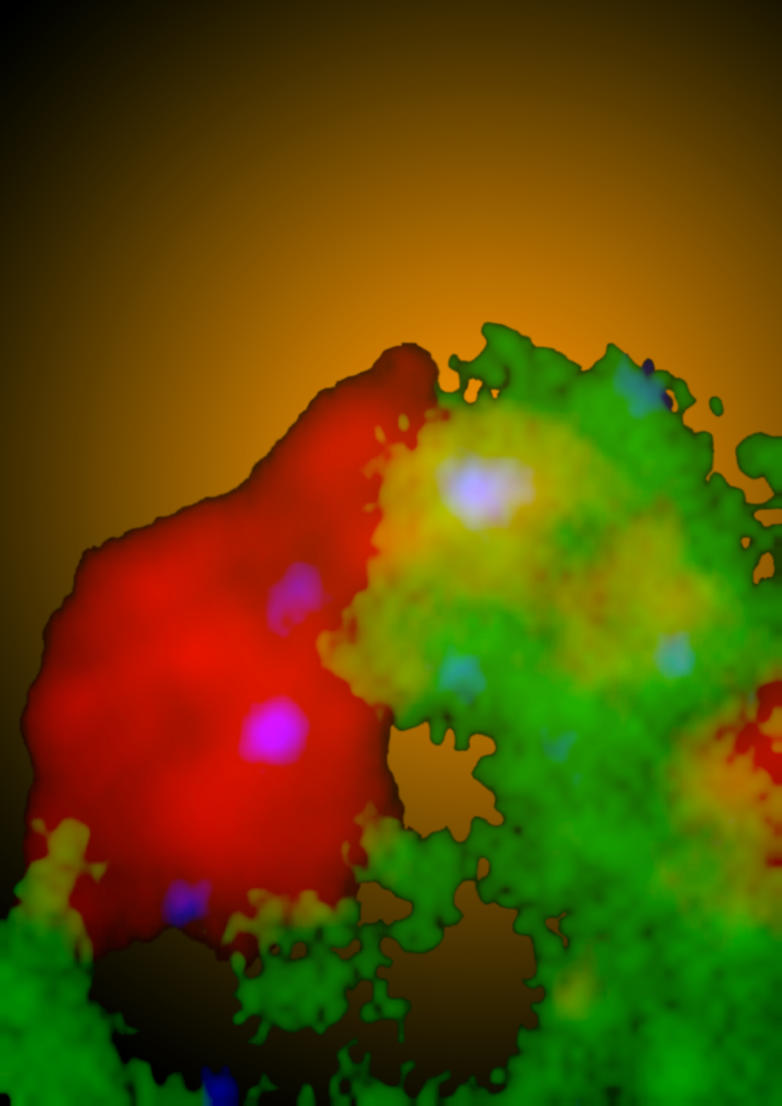
Two different human chromosomes “painted” in green and red are shown to intermingle in the yellow area.

