# Correction: Interphase Chromosomes Mingle with Their Peers

**DOI:** 10.1371/journal.pbio.0040217

**Published:** 2006-04-25

**Authors:** Françoise Chanut

In *PLoS Biology*, volume 4, issue 5: DOI: 10.1371/journal.pbio.0040174

The caption of the image should be “Transcription foci/factories (in blue) are found within areas of intermingling between Chromosome 3 (in red) and the remaining genome (in green), and may mediate functional interactions between them.”

